# Cell Counting in Human Endobronchial Biopsies - Disagreement of 2D versus 3D Morphometry

**DOI:** 10.1371/journal.pone.0092510

**Published:** 2014-03-24

**Authors:** Vlad A. Bratu, Veit J. Erpenbeck, Antonia Fehrenbach, Tanja Rausch, Susanne Rittinghausen, Norbert Krug, Jens M. Hohlfeld, Heinz Fehrenbach

**Affiliations:** 1 Department of Pneumology, Philipps-University Marburg, Universities of Giessen and Marburg Lung Center (UGMLC), Member of the German Center for Lung Research (DZL), Marburg, Germany; 2 Fraunhofer Institute of Toxicology and Experimental Medicine (ITEM), BREATH, Member of the German Center for Lung Research (DZL), Hannover, Germany; 3 Department of Respiratory Medicine, Hannover Medical School, Biomedical Research in Endstage and Obstructive Lung Disease Hannover (BREATH), Member of the German Center for Lung Research (DZL), Hannover, Germany; Institute of Lung Biology and Disease (iLBD), Helmholtz Zentrum München, Germany

## Abstract

**Question:**

Inflammatory cell numbers are important endpoints in clinical studies relying on endobronchial biopsies. Assumption-based bidimensional (2D) counting methods are widely used, although theoretically design-based stereologic three-dimensional (3D) methods alone offer an unbiased quantitative tool. We assessed the method agreement between 2D and 3D counting designs in practice when applied to identical samples in parallel.

**Materials and Methods:**

Biopsies from segmental bronchi were collected from healthy non-smokers (n = 7) and smokers (n = 7), embedded and sectioned exhaustively. Systematic uniform random samples were immunohistochemically stained for macrophages (CD68) and T-lymphocytes (CD3), respectively. In identical fields of view, cell numbers per volume unit (N_V_) were assessed using the physical disector (3D), and profiles per area unit (N_A_) were counted (2D). For CD68^+^ cells, profiles with and without nucleus were separately recorded. In order to enable a direct comparison of the two methods, the zero-dimensional CD68^+^/CD3^+^-ratio was calculated for each approach. Method agreement was tested by Bland-Altmann analysis.

**Results:**

In both groups, mean CD68^+^/CD3^+^ ratios for N_V_ and N_A_ were significantly different (non-smokers: 0.39 and 0.68, p<0.05; smokers: 0.49 and 1.68, p<0.05). When counting only nucleated CD68^+^ profiles, mean ratios obtained by 2D and 3D counting were similar, but the regression-based Bland-Altmann analysis indicated a bias of the 2D ratios proportional to their magnitude. This magnitude dependent deviation differed between the two groups.

**Conclusions:**

2D counts of cell and nuclear profiles introduce a variable size-dependent bias throughout the measurement range. Because the deviation between the 3D and 2D data was different in the two groups, it precludes establishing a ‘universal conversion formula’.

## Introduction

Airway inflammation is a characteristic feature of chronic airway diseases like asthma and chronic obstructive pulmonary disease (COPD). Studies aiming at unravelling the pathophysiological mechanisms of these entities or at the clinical evaluation of drugs with anti-inflammatory or disease-modifying activity require the implementation of techniques for the reliable quantification of the inflammatory and/or ‘inappropriate remodelling’ processes of the airways [1]–[5]. In clinical studies, endobronchial biopsies offer a suitable gateway to the assessment and quantification of such processes related to the airway mucosa. As the inflammatory phenotype may differ between the lumen of the airways (sampled by bronchoalveolar lavage (BAL)), the epithelium and the lamina propria (both sampled by biopsy) [6], [7], the quantitative morphologic study of endobronchial biopsies provides valuable data, which cannot be obtained from BAL, sputum analysis, or exhaled breath condensates.

Many attempts have been made to standardise all steps of the procedure, including sampling of the airway tree, excision, processing and sampling of the specimen and analysing the histology [2],[8]–[11]. The standard practice of counting the number of cut cell profiles of interest in a tissue section and normalising these counts to submucosal area or to length of the epithelial reticular basement membrane (i.e., a two-dimensional (2D) design) continues to be a widely used quantitative approach. For theoretical reasons, the probability of visible cells being counted in a 2D section is not only proportional to the cell density, the variable of interest, but also to their size and the orientation relative to the section plane, as well as to the section thickness, thus introducing a bias in favour of larger cells. Design-based stereology offers tools, such as the disector and the fractionator, to count 3D particles in microscopy (i.e., cells or alveoli) without the need for any bias-prone assumptions about their geometry, orientation, and distribution [12]. The importance of implementing design-based stereologic approaches into quantitative studies of lung structures including biopsies was highlighted by an official research policy statement of the ATS/ERS [13], which recommends the disector as the gold standard for counting of 3D particles, such as cells.

Whereas the general advantages and disadvantages of 3D versus 2D approaches were discussed elsewhere [2], the present study addresses the issue of statistical agreement between the data obtained by two different quantitative methods: an unbiased stereological numerical density estimator, i.e. the physical disector, and the classical 2D approach of counting cell or nuclear profiles per area unit. To investigate the robustness of the method agreement we used Bland-Altman analysis to investigate two groups of human subjects: non-smokers and smokers, which displayed differences in the inflammatory phenotype in previous biopsy studies.

We further describe an experimental design for the analysis of endobronchial biopsies, which allows obtaining multiple section series from one biopsy, in accordance with the principles of systematic uniform random sampling. Thus, in a given study several section series, each of them representative of the whole biopsy, can be obtained and assigned to different histochemical or immunohistochemical stainings.

## Materials and Methods

### Subjects

In this study we investigated endobronchial biopsies from 7 healthy non-smokers and 7 smokers. None of the included subjects suffered from acute bronchitis within 4 weeks before the investigations. All subjects were volunteers who gave their written consent after being fully informed about the purpose and nature of the investigations. This study was approved by the ethics committee of Hannover Medical School (Hannover, Germany).

### Bronchoscopy

The subjects received premedication according to the routine protocols: 0.2 mg aerosolized salbutamol, fractionated intravenous midazolam (0.05 mg/kg) and 3 ml nasal topical lidocaine 4%. The healthy non-smokers underwent inhalative bronchial anaesthesia with 2.5 ml lidocaine 4% by electronically controlled and regulated inhalation using the AKITA® inhalation system, while the smokers received local anaesthesia of the bronchial mucosa during the bronchoscopy using lidocaine 2% up to a maximal dose of 6 mg/kg as previously described [14]. Differences in bronchial anaesthesia were due to answering another research question, which was not part of this study or likely to impact on its results. During flexible bronchoscopy performed according to the international guidelines [11], [15] two or three biopsies per subject were collected from the segmental branches of the right lower pulmonary lobe using the fenestrated cup Radial Jaw® biopsy forceps (Boston Scientific Medizintechnik GmbH, Ratingen, Germany).

### Biopsy Processing and Sampling

**Figure 1:**
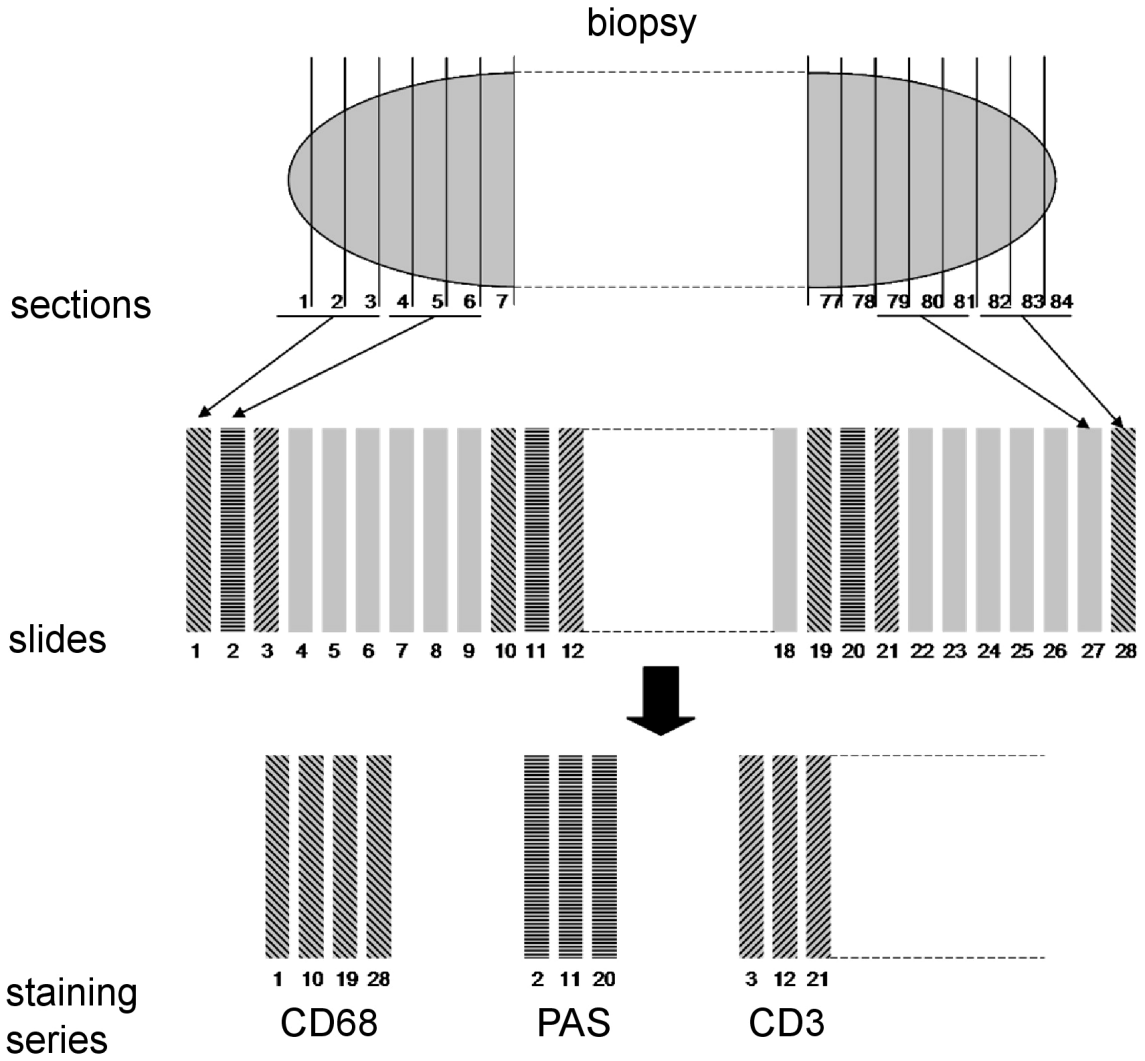
Schematic SUR sampling of the sections of a biopsy. After exhaustive sectioning, every three sections were mounted on numbered glass slides (1 to 28 in this example). With a random outset between the 1^st^ and the 9^th^ slide, nine slide samples, each consisting of every 9^th^ glass slide, were collected and stained.

The collected biopsies underwent fixation in 4% phosphate-buffered formaldehyde overnight. After transfer into 2% aqueous agarose, the biopsies were embedded in paraffin wax. The paraffin blocks were exhaustively sectioned using a motorized rotary microtome (HM355S, Microm International GmbH, Walldorf, Germany) with a 2-μm average block advance (BA), calibrated by means of a digital calliper measuring the block height before and after cutting 500 sections at a given microtome setting. Every three consecutive sections were mounted on numbered glass slides. The contribution of the variation between biopsies of the same airway generation to the total variability is very low [16], in fact much lower than the usually attainable precision of the quantitative estimators, so that only the biopsy yielding the most sections/slides was selected from each subject for investigation. According to the fractionator and systematic uniform random sampling (SURS) principles [17], [18], every 9^th^ or 20^th^ slide, depending on the size of the biopsy, was sampled in a slide series with a random outset between the 1^st^ and the 9^th^ or the 20^th^ slide of a biopsy, respectively (Figure 1). This resulted in a section-sampling fraction of 1/9 or 1/20, respectively. By this algorithm two samples of 5–11 glass slides were collected for indirect immunohistochemistry. Besides complying with the stereological principles of SURS, the number of collected sections is also in accordance with the findings of previous investigations regarding the between-section variability of endobronchial biopsies [19].

### Indirect Immunohistochemistry

The collected samples were used to identify T-lymphocytes and macrophages, respectively: one sample was stained for CD3^+^ (polyclonal rabbit anti-human 1∶100, DAKOCytomation, Glostrup, Denmark) and the other for CD68^+^ (monoclonal mouse anti-human PG-M1 1∶100, DAKOCytomation) cells as previously described [20].

### Computer-assisted 2D and 3D Quantification of Inflammatory Cells

All cell counts were conducted on a computer-linked Olympus BX 51 light microscope equipped with a motorized stage and the CAST-Grid 2.01 system (Olympus, Ballerup, Denmark) using oil immersion lenses. The final magnifications were 1,400×(CD68^+^) and 2,100×(CD3^+^) with a numerical aperture setting of 1.00 and 1.40 respectively, in order to minimize the depth of field. The reference compartment was confined to the lamina propria of the airway mucosa for both cell types. The stained T-lymphocytes and macrophages were quantified over the entire sample by performing the 2D and 3D counting simultaneously.

#### 3D Counting – The Physical Disector

**Figure 2:**
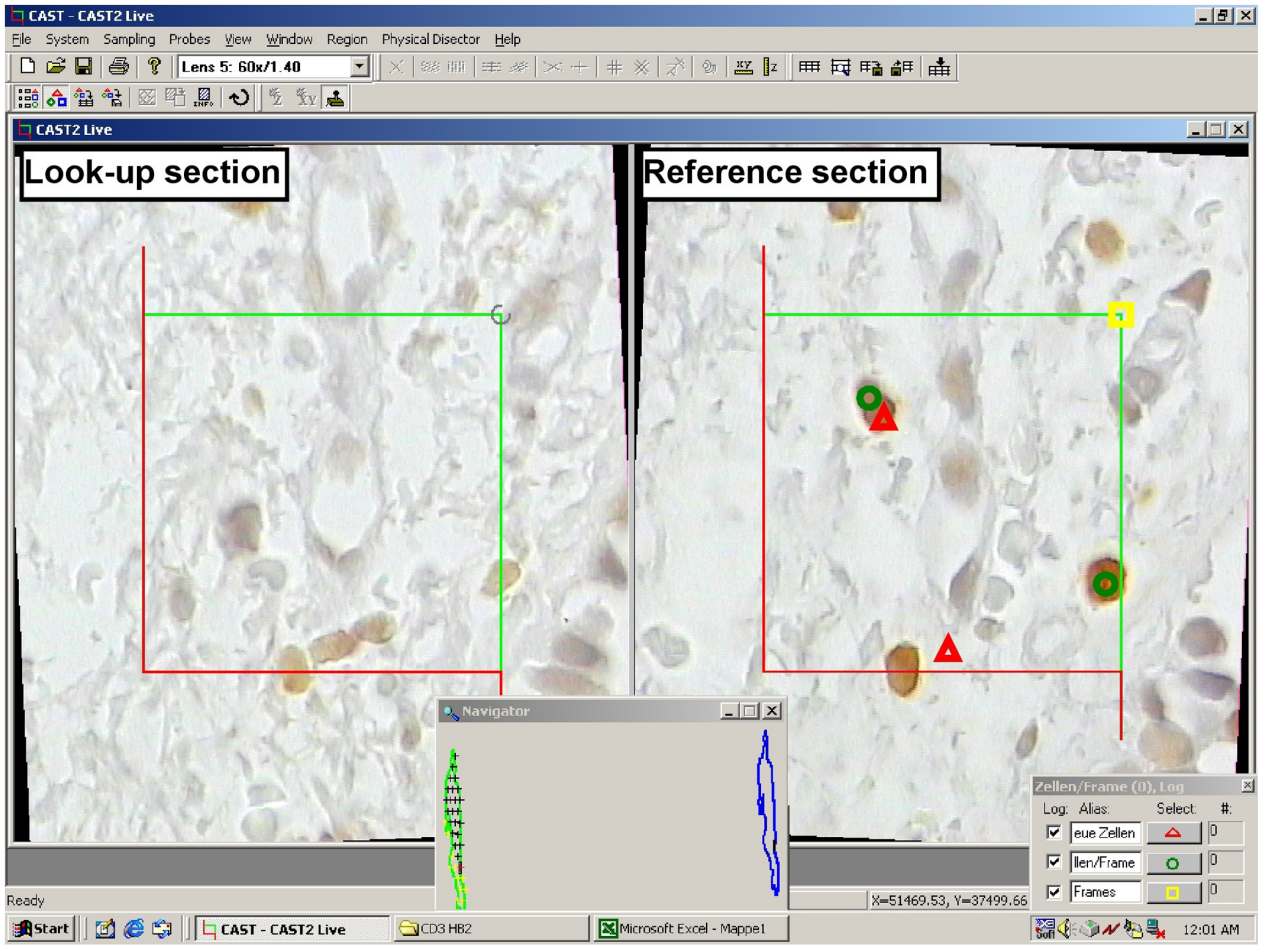
Physical disector (3D) and profile counting (2D) within a consecutive reference and look-up section. Red triangles mark cell profiles seen in the reference section which are not present in the look-up section (bidirectional counting); green circles mark all cell profiles seen in the right section; yellow squares mark each assessed counting frame/field of view. The cell profile cutting the lower exclusion (red) line is not counted either in 3D or in 2D.



For 3D counting, the physical disector was used by analysing two consecutive sections: a reference and a look-up section [13], [17], [21], the disector height thus being equal to the section thickness (2 μm). The choice of the disector pair from the three sections mounted on each slide was based on the technical quality of the specimens. A representative SUR sample of physical disectors spaced at 54 or 120 μm over the entire biopsy was analysed. For each disector, SUR pairs of registered fields of view were sequentially presented on the high-resolution monitor and positively stained cell transects within a single focal plane were sampled and assessed with an unbiased counting frame [17], [22], with an area of 30% of the displayed field of view. Only profiles of those cells were counted in the reference section that did not touch the exclusion lines of the unbiased counting frame and were not present in the look-up section (Figure 2). In order to increase efficiency the counting was performed bidirectionally by interchanging the reference and the look-up sections, as generally recommended [17]. Area-sampling fractions ranging 4-16% for the anti-CD3 and 9–25% for the anti-CD68 stained sections yielded sufficiently high counts per biopsy to achieve appropriate coefficients of error [18], [23]. The number of cells per volume unit, the numerical density (N_V_), was estimated for each biopsy and cell type according to:

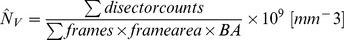



#### 2D Counting – The ‘Area Profile' Approach

The 2D profile counting was performed on one of the two sections, on the same fields of view sampled for 3D counting (Figure 2). The counting criterion for the small T-lymphocytes with poorly developed cytoplasm was the stained cell profile. For quantifying macrophages two 2D approaches were used by counting: 1) all stained cell transects (with or without nucleus) and 2) only stained transects containing a nuclear profile – in order to reduce the influence of differing cell size, while assuming that nuclear size varies less [2]. The results were recorded as cumulative counts for each section. The number of profiles per area unit (N_A_) was estimated for each biopsy and cell type according to:
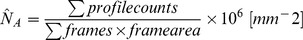



### Statistical analyses

#### Descriptive Statistics

For each subject and selected biopsy, N_V_ [mm^−3^] and N_A_ [mm^−2^] were calculated as discrete values accompanied by the coefficients of error (CE) calculated with the quadratic approximation formula (data not shown), which takes into account the nugget effect, i.e. the discontinuous distribution of cells, which tend to form clusters rather than being randomly distributed [23]–[25]. Mean values are accompanied by the mean CE (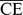), calculated as the quadratic mean of the individual CEs.

The observed variance (OV) of the estimates has two contributions: (i) the inherent variation between the individuals (biological variability) and (ii) the variation introduced by the employed sampling scheme, which is depicted by 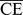. To ensure that OV depends mainly on the biological variability, the design had to be tuned so that the variation introduced by the sampling was smaller than the biological variation.

The two cell counting methods deliver results with different physical dimensions (mm^−3^ and mm^−2^ respectively) and very different magnitudes. To allow for a direct comparison of the 3D and 2D approach only, zero-dimensional ratios between the densities of two cell populations were calculated using each method. To avoid the pitfall of a potential size-bias similarly affecting both terms of the ratio, two cell populations with clearly different mean sizes were investigated: macrophages and T-lymphocytes. The mean ratio values are reported for each group of subjects. The CEs of the ratios (CE_r_) were calculated as the square root of the sum of squared CEs of the ratio terms. Mean ratios are accompanied by mean CE_r_ (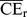), calculated as the quadratic mean of CE_r_.

#### Inferential Statistics and Exploratory Data Analysis

All statistical analyses were performed using SigmaStat 3.1 (Jandel Scientific, Erkrath, Germany). The Kolmogorov-Smirnov test was used to verify the data for a normal distribution. The equality of variances was tested by the variance ratio test (F-test). Parametric testing was then applied to data drawn from normally distributed populations with equal variances. Otherwise, non-parametric tests were employed. Pearson's correlation coefficient (r) was used to test the relationship between 3D and 2D density estimates. For each group of subjects, each of the 2D approaches and the physical disector design were tested for differences of the mean CD68^+^/CD3^+^ ratios using Wilcoxon's signed rank test. The mean CD68^+^/CD3^+^ ratios obtained by 2D cell profile counting were tested for differences between the two groups by Mann-Whitney's non-parametric rank sum test, after standardisation by dividing them by the corresponding 3D mean ratios. *p* values<0.05 were considered to be significant.

The method agreement was tested for interchangeability of the results using the Bland-Altman analysis [26], [27]. Besides the inherent random measurement error of each method, a systematic error, i.e. bias, of one or both methods can lead to significant discrepancies in the results. The bias can be either constant (on offset) or proportional to the measurement magnitude. Based on theoretical reasons, we regarded the physical disector as the standard method and the area profile approach as the alternative method. Spearmann's rank correlation coefficient was used to assess the relation between the ratio differences of the two designs and their mean values. The mean of the differences, i.e. the bias, was modelled as a function of the magnitude of the measurement by linear regression. The limits of agreement were then obtained from the regression function ±2S_y|x_ (standard error of the estimate), in a manner similar to the definition of the 95% limits of agreement [27], [28]. To be acceptable, the 95% limits of agreement had to lie within ±2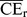 for each group. This takes into account the precision of the ratio estimators, as quantified by the mean CE. The regression coefficients and the intercepts for the two groups were tested for a significant difference by Student's t test [29]. *p* values<0.05 were considered to be significant. For this threshold of type I error, the desired statistical power was >80%.

## Results

### Subjects

The subjects' demographic and clinical data are shown in Table 1. In the non-smoker group, six subjects were never-smokers, whereas one subject was ex-smoker with a history of 0.9 packyears and had quit more than 1 year before the onset of the study. All non-smokers had normal spirometry results, no signs of obstructive pulmonary disease and were therefore designated as ‘healthy’. All smokers were actively smoking at the time of enrolment. In this group 3 subjects (2 males, 1 female) had normal FEV_1_/FVC ratios; the other 4 subjects (2 males, 2 females) had FEV_1_/FVC ratios<70% (58.1%–66.8%) and were diagnosed with COPD stage 1 according to the GOLD criteria [30], [31].

**Table 1:** Subject Demographics.

Group	Non-smokers	Smokers
**No. of subjects**	7	7
**Sex (M/F)**	4/3	4/3
**Age (years)**		
Mean ± SD	30.9±6.96	46.7±7.91
Range	25–42	40–61
**FEV_1_ (L)**		
Mean ± SD	4.6±0.59	3.4±0.96
Range	3.80–5.43	2.35–4.69
**FEV_1_/FVC (%)**		
Mean ± SD	81.7±2.61	68.5±9.2
Range	78.8–86.3	58.1–80.2
**Subjects with airway obstruction**	0/7	4/7
**Packyears**		
Median	0	33
Range	0.0–0.9	23.4–54.4

### Comparison of 2D and 3D Inflammatory Cell Counts

**Figure 3:**
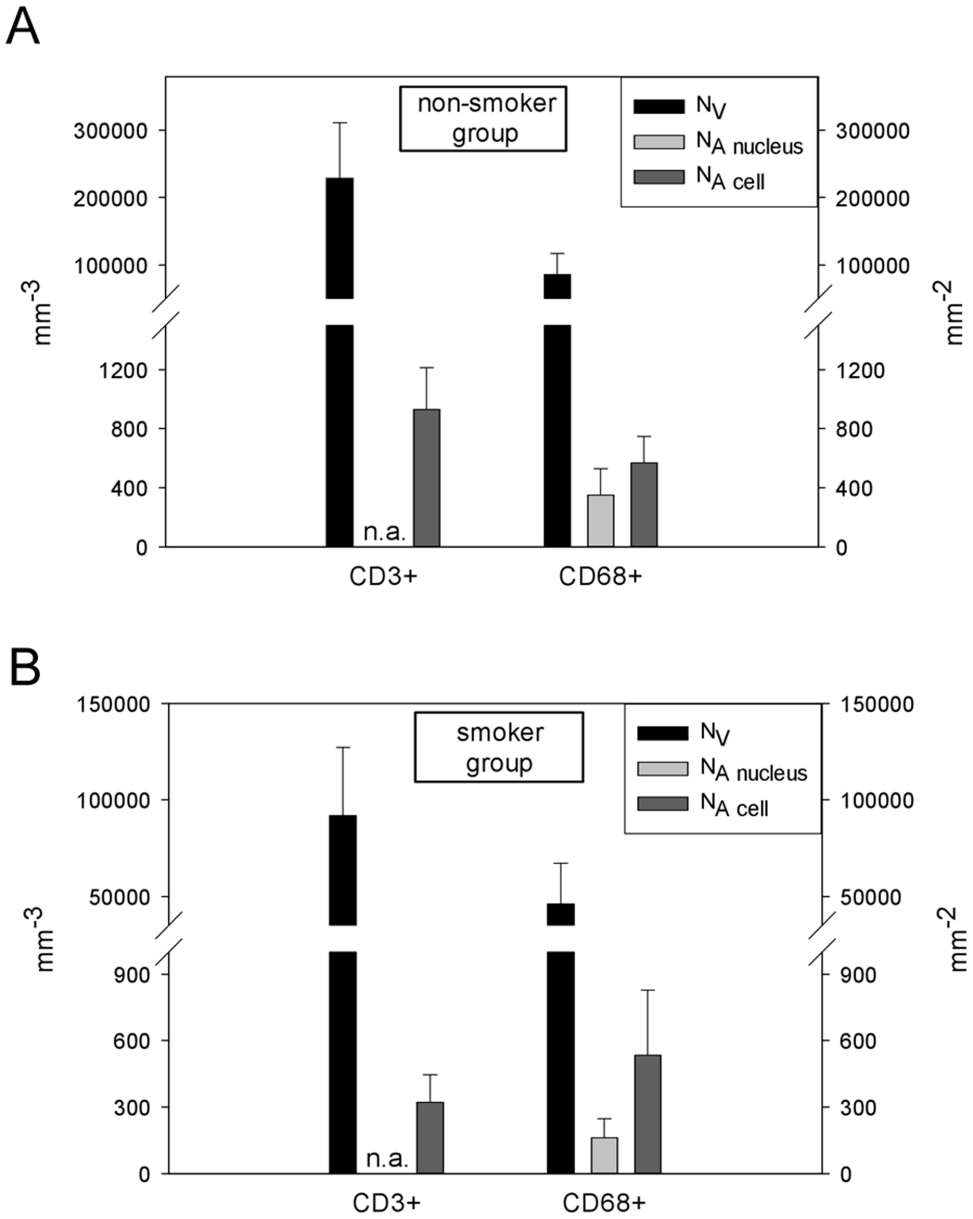
Mean counts per unit volume and area (mean + SD) by group and cell population.

Table 2 shows mean counts per unit for each group, cell population and counting method. The area profile number was considerably higher when counting all cell profiles instead of only nuclear profiles in both groups. The coefficients of variation (CV) of the 2D and 3D densities ranged from 29% to 51%. Although the counting was performed on the same fields of view, the relative variation between subjects tended to be lower in the 3D than in the 2D approach (Figure 3). The 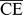 were fairly constant (6.6 to 12.4%) regardless of the approach used, the cell population under investigation or the study group. They represented 1.7–10.9% of the observed variation (OV), in accordance with the recommendation for the variance of the estimator (i.e. counting noise) to be less than half of the OV [24], [32].

**Table 2:** Quantitative Morphological Data by Group and Cell Type.

Group	Cell Type	N_V_ (mm^−3^)		N_A nucleus_ (mm^−2^)		N_A cell_ (mm^−2^)	
		Mean	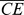	mean	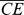	mean	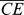
non-smokers	CD68^+^	85987	9.7%	350	10.1%	569	7.6%
	CD3^+^	228612	9.3%	N. A.	N. A.	931	9.3%
smokers	CD68^+^	46025	11.5%	163	12.4%	534	6.6%
	CD3^+^	91870	10.4%	N. A.	N. A.	322	11.2%

*Definition of abbreviations*: N_V_  =  numerical density, N_A nucleus_  =  nuclear profile per unit area, N_A cell_  =  cell profile per unit area, 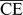  =  coefficient of error of the mean estimate, N. A.  =  not analysed

**Figure 4:**
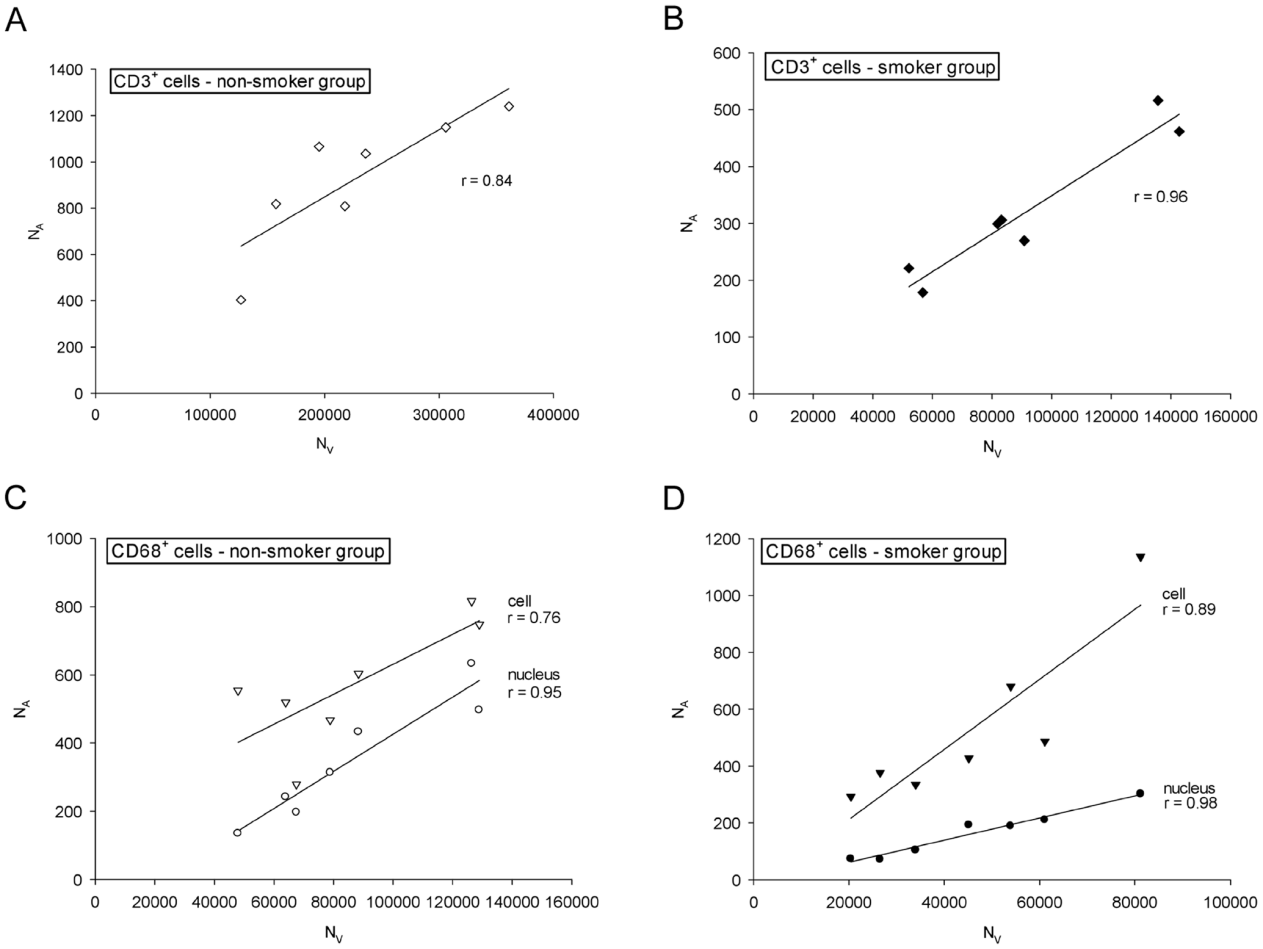
2D profiles per unit area *versus* 3D numerical density. (a) T-lymphocytes, non-smokers, r = 0.84, *p* = 0.017; (b) T-lymphocytes, smokers, r = 0.96, *p*<0.001; (c) macrophages, non-smokers, r_nucleus_ = 0.95, *p* = 0.001; r_cell_ = 0.76, *p* = 0.046; (d) macrophages, smokers, r_nucleus_ = 0.98, *p*<0.001; r_cell_ = 0.89, *p* = 0.007

**Figure 5:**
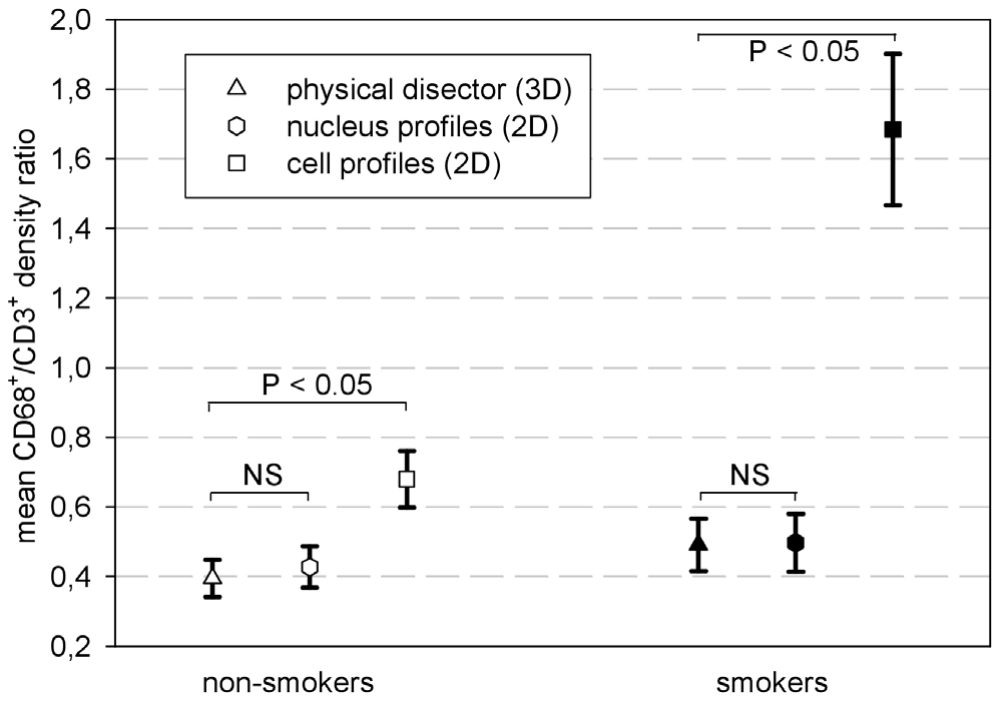
Mean CD68^+^/CD3^+^ cell density ratios (mean ± SEM) for each design and study group.

**Figure 6:**
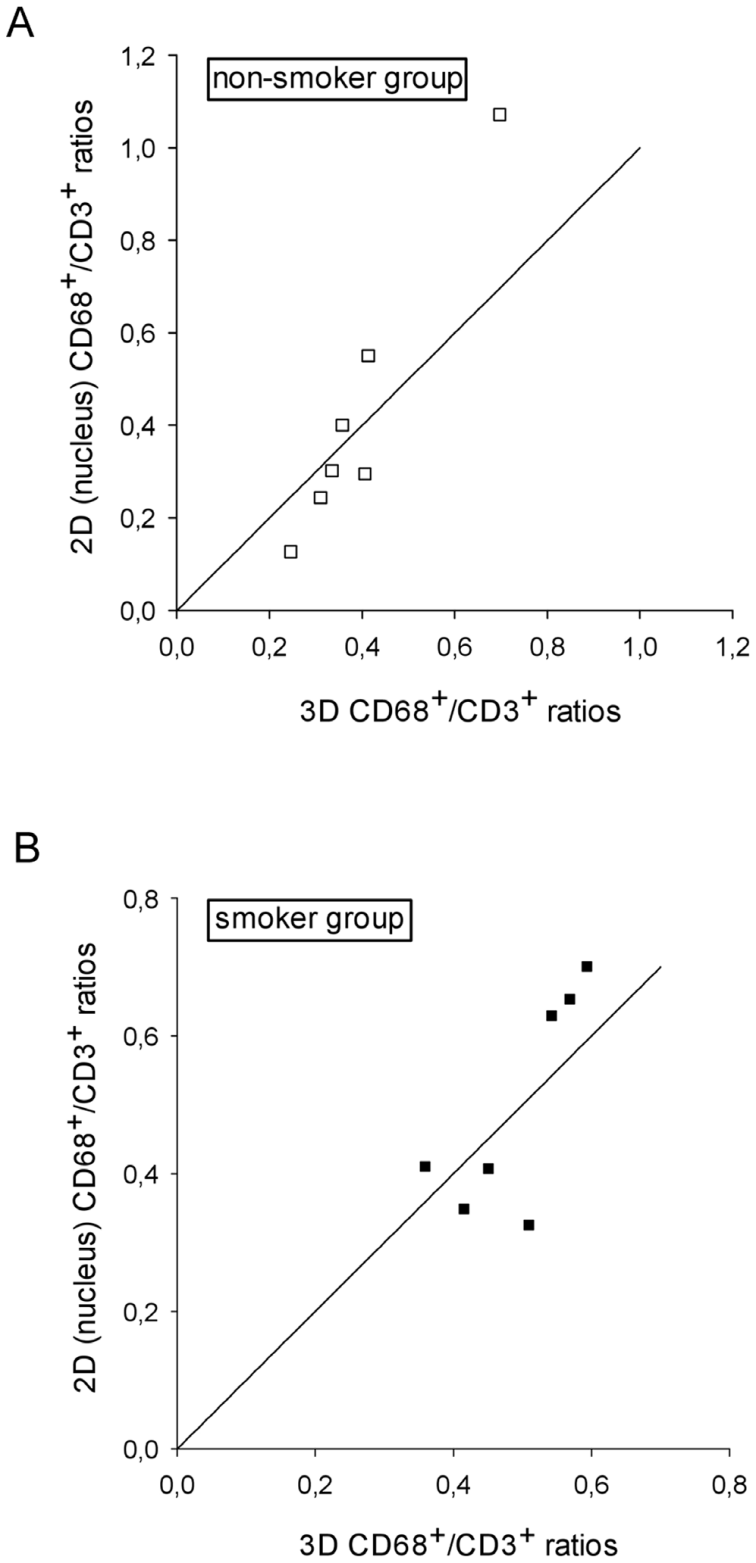
CD68^+^/CD3^+^ cell density ratios estimated by the 2D (nucleus) and 3D design for the non-smoker, r = 0.97, p<0.001 (a) and smoker, r = 0.77, p<0.05 (b) group with the line of equality (y = x).

In both study groups, N_A_ and N_V_ were very strongly and significantly correlated for both T-lymphocytes (Figures 4a and 4b) and macrophages (Figures 4c and 4d), respectively. The calculated slopes of the regression lines ranged 0.0029 to 0.0123. Because the 2D data were recorded as cell or nuclear profile counts per area unit (N_A_), whereas in the 3D approach cell numbers per volume unit (N_V_) were obtained, different scale units precluded direct statistical testing of the differences or the agreement between these methods. To overcome this problem the dimensionless ratio between CD68^+^ and CD3^+^ counts was calculated by each approach. The 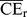 ranged from 12 to 16.7% (Table 3). In each study group, the mean CD68^+^/CD3^+^ ratios obtained from 3D and 2D cell profile counts showed statistically significant differences (*p* = 0.016), with 2D values being 1.7 and 3.4 times higher for non-smokers and smokers respectively. This difference in the relative amplitude of the 2D estimator across the two subject groups was also statistically significant (*p* = 0.002). When counting only CD68^+^ cell profiles containing a nucleus, the mean results of the 3D and the 2D nuclear profile approaches were very similar and the level of significance was not reached: non-smokers *p* = 0.938, smokers *p* = 0.688 (Figure 5). Nevertheless, after plotting the ratios calculated from the 2D nucleus and the 3D design against each other, it is fairly obvious that most measurement pairs are not in good agreement, i.e. they were widely scattered around the line of equality y = x (Figure 6).

**Table 3:** CD68^+^/CD3^+^ Cell Ratios by Group and Counting Design.

Group	CD68^+^/CD3^+^		CD68^+^/CD3^+^		CD68^+^/CD3^+^	
	3D		2D nucleus		2D cell	
	Mean	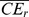	Mean	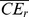	Mean	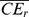
non-smokers	0.39	13.4%	0.43	13.7%	0.68	12.0%
smokers	0.49	15.5%	0.50	16.7%	1.68	12.9%

*Definition of abbreviations*: 3D  =  physical disector, 2D nucleus  =  counts of nuclear profiles, 2D cell  =  counts of cell profiles (with and without nucleus), 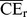  =  coefficient of error of the mean ratio estimate.

**Figure 7:**
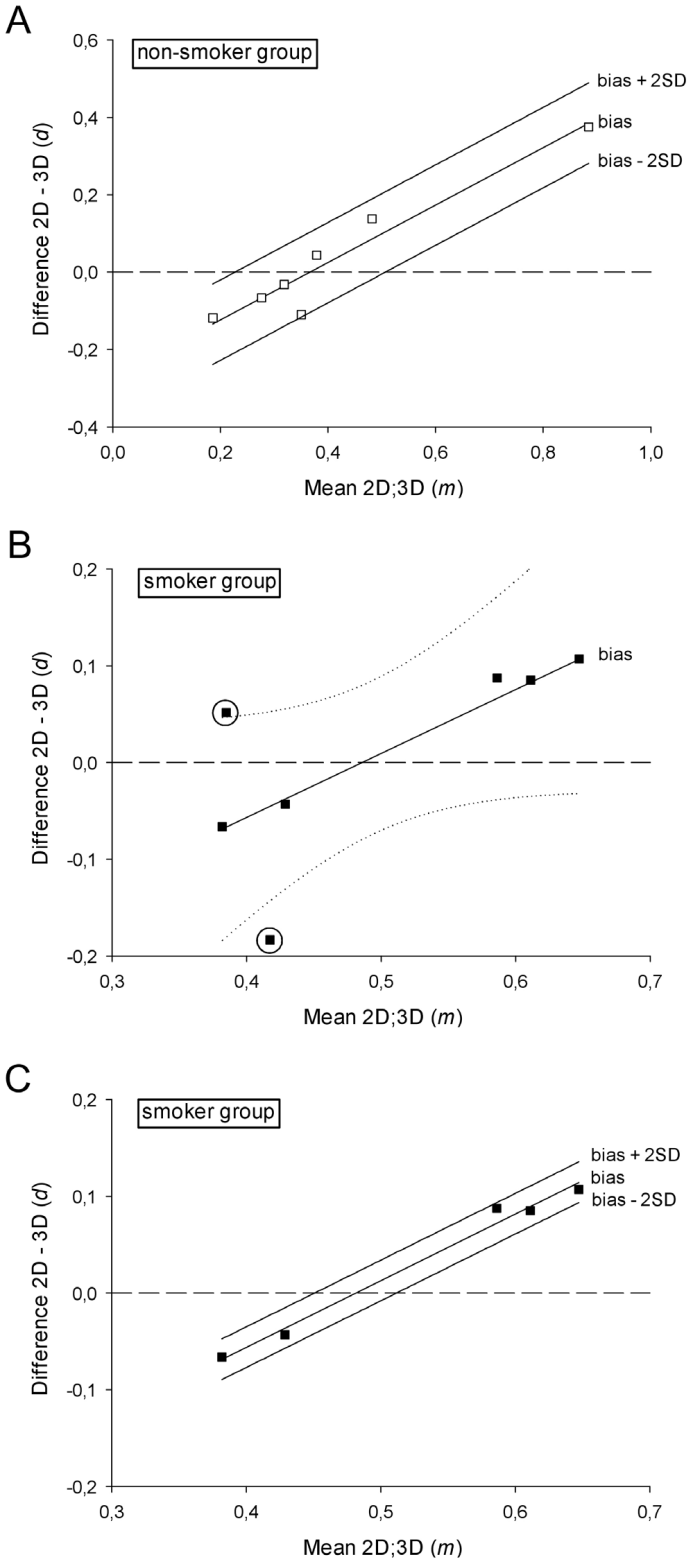
A clear correlation between the difference and the mean magnitude can be noticed for both groups. Dashed line y = 0 represents the line of equality, which stands for perfect agreement. (a) Regression based mean difference (bias) and 95% limits of agreement for the differences of the CD68^+^/CD3^+^ cell density ratios as determined by the 2D nucleus and 3D approaches in the non-smoker group. All values lie within the interval between the calculated 95% limits of agreement; (b) Regression based mean difference (bias) with 95% C.I. of the regression line (dotted) for the differences of the CD68^+^/CD3^+^ cell density ratios in the smoker group. The 95% C.I. includes several horizontal lines (slope = 0) so that the fitted linear model does not achieve the desired statistical significance. Two large outliers encircled; (c) Regression based mean difference (bias) and 95% limits of agreement for the differences of the CD68^+^/CD3^+^ cell density ratios as determined by the 2D nucleus and 3D approaches in the smoker group after removing the two large outliers. All values lie within the interval between the calculated 95% limits of agreement. Notice the similar slope to the fitted model in Figure 7a (non-smoker group).



The agreement was assessed by plotting the differences between the ratios from the two approaches against their mean (i.e. magnitude) for each subject (Figure 7) [33], [34]. A striking relation between the difference and the magnitude was noticed: r_s_ = 0.89 for the non-smoker group and r_s_ = 0.79 for the smoker group, both statistically significant (non-smoker *p*_r_<0.001, smoker *p*_r_ = 0.025). In the non-smoker group, the ratio means reflect 91% of the variability in the ratio differences, as measured by the coefficient of determination r^2^. The differences between the two methods tended to be negative for low magnitudes and positive for high values. The linear regression of the differences (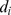) on the magnitudes (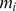) gave the proportional bias of the 2D ratios compared to the 3D approach (Figure 7a, Eq.1):

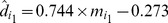
Since S_y|x_ = 0.053 the regression based 95% limits of agreement were 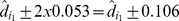.

This falls under the criteria of acceptance for the 95% limits of agreement set to 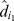
±2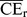, i.e. 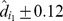. The power of the performed regression was 97.6%, thus indicating a high appropriateness in describing the relationship between the difference and the magnitude.

In the smoker group fitting a linear regression model showed that the ratio means account for only 41.4% of the variability of the ratio differences, as measured by the coefficient of determination r^2^. Regarding the regression equation, the chosen level of significance was reached neither for the slope (regression coefficient), nor for the analysis of variance (F-test): *p* = 0.071 (Figure 7b). The statistical power of the performed regression for the sample size n = 7 and α = 0.05 was 43.4%. Two large outliers (encircled in Figure 7b) had very low CD68^+^ and CD3^+^ N_V_ (the lowest in our sample) and therefore high CE and CE_r_. Because this very high measurement error is likely to be a strong confounder in a sample of n = 7, we excluded these two subjects and then repeated the regression analysis of the differences on the means. This led to a remarkable improvement of the fitted model, with the mean ratios reflecting 98.3% of the variability in the ratio differences. The regression equation of 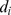 on 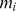 became:
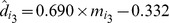
which represents the proportional bias of the 2D ratios compared to the 3D approach (Figure 7c). Because of the decrease in 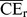 to approximately 11% we redefined acceptable agreement as 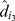
±0.11. We used S_y|x_ = 0.011 to calculate the regression based 95% limits of agreement as 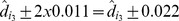. These limits of agreement fully comply with the redefined acceptable agreement. The statistical power of the regression increased considerably to 98.1% (for α = 0.05).

The equations were tested to see if the regression follows the same model in both study groups. The difference between the regression coefficients of Eq. 1 and 2 was not statistically significant: 95% C.I. [−0.396; 0.504]. The common (or weighted) regression coefficient was computed: b_c_ = 0.736. The two intercepts of Eq. 1 and 2 showed a statistically significant difference (*p*<0.01). Thus Eq. 1 and Eq. 2 became 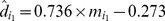 and 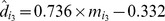, respectively.

## Discussion

Endobronchial biopsies have been widely used for quantitative assessments of inflammation and the related structural changes in chronic inflammatory airway diseases [8]–[10], [16], [35]–[44]. Most biopsy studies rely on 2D counting of inflammatory cells [2], although 3D approaches are readily available for more than 20 years [21]. Design-based stereology represents *the state of the art* in other biomedical research areas, such as neurosciences and nephrology [45], [46], whereas its implementation in pulmonary research as a standard quantitative technique has been approached only recently [47]. The official research policy statement of the ATS/ERS in particular recommends using the disector as gold standard for the counting of 3D particles, such as cells [13]. Whereas stereological approaches have been considered time-consuming in the past [2], with the integration of automated whole slide scanners, automated section sampling, computer-assisted measurements, and automated capture and registration of physical disectors this no longer holds true today [48]; [49]. One other study compared the data obtained by design-based stereological and assumption-based ‘area profile’ counting techniques [38]. However, the correlation analysis employed therein to assess the agreement between the two approaches is insufficient, as it only demonstrates more or less linear variation of the data, but not their equality [26], [33].

The present study addresses the issue of agreement between the data supplied by the widely used 2D cell or nuclear profile counting and those relying on 3D cell counts. Because size and its variation are thought to be a major source of bias [2], [50], two cell populations (‘small’ T-lymphocytes and ‘large’ macrophages) were quantitatively assessed using both approaches in parallel in endobronchial biopsies of two human subject groups. The rationale for including two groups was to analyse the robustness of the assessed agreement and characterise its variability. It has to be emphasized that our study did not pursue a direct quantitative comparison of the inflammatory phenotypes of the two groups, as they were recruited in two different settings, or drawing any conclusion about the differences therein and their potential biological significance.

### Coefficients of error of 2D and 3D design are comparable

Prior to assessing the accuracy, quantified by the systematic error or bias, one should demonstrate adequate precision, quantified by the random measurement error. The estimated CE (inherent counting noise) for the 2D and 3D densities were acceptable with regard to the biological variability of the samples [24], [32]. They were also very similar to previously published results on the precision of 2D counting for different cell populations, which quoted CE in the range of 2–11% [7], [44], [51]. However, the interpretation of quantitative results from bronchial biopsies poses certain challenges and their advantages are offset by the large variability between and within patients. This in turn may reduce the reliability of the estimates. The large observed coefficients of variation of each group in this study were consistent with the rather scarce previous findings in 2D counting designs [2], [16], [38]. In general, the relative contribution of the variation between individuals, tissue blocks, fields of view, and measurements, to the total biological variation was assessed earlier [52]. It was demonstrated that measurements and fields of view account for only 3% and 8% of the total variance, respectively. Whereas the level of individuals accounts for 70% of the total biological variation [52]. Therefore, the official research policy statement of the American Thoracic Society/European Respiratory Society pointed out that “the general rule is that the “noise” should not exceed the “signal,” CE^2^(method)≤0.5 CV^2^(biological), and efficiency considerations means that it is wasteful of resources to make CE(method) << CV(biological)” (i.e., the “do more less well” paradigm)” [13]. These aspects related to biological variability can be addressed through a rigorous study and sampling design [8]. The adopted SUR sampling, which included 5–11 section pairs per biopsy and many fields per section, efficiently controlled the within-biopsy variability, adhering to the results of previous 2D counting studies [19], [53].

The counted entities were bidimensional cell transects in one case and three-dimensional cells in the other case. The two designs delivered results with very different orders of magnitude, mostly 10^2^ for 2D and 10^4^–10^5^ for 3D counts, and expressed in different scale units: mm^−2^ and mm^−3^ respectively. This is an inherent problem in biopsy research, which has to rely mostly on cell densities, as the reference volume is not known and therefore no absolute cell numbers can be derived. Caution is necessary in the interpretation of density data in order to avoid the ‘reference trap’, when the unknown reference volume is prone to different changes during pathophysiological processes or tissue processing and thus alters the density values without any change in the absolute quantities.

### Correlation and regression analysis are not appropriate assessment tools of agreement

It is obvious that the two data sets cannot substitute each other, although they display very strong positive correlations (Figure 4), similar to previously published biopsy data for other inflammatory cells of the airways [38]. This is not surprising, as scale units do not affect correlation and it would be quite surprising if two methods designed to quantify the *same* underlying structural entity were not correlated. In our case the relationship between N_A_ and N_V_ is described by the mean cell height perpendicular to the section plane [38], [54]–[56]. Nonetheless, this does not imply good agreement, as correlation lacks sensitivity to bias [33], [57]. In addition, the agreement of two methods would require the slope of the regression line as plotted in Figure 4 to be approximately 1, taking into account the random measurement error of both methods [58]. Although all four graphs demonstrate good to very good correlation, the slopes are 0.0029 to 0.0123, which is far from the line of equality (slope = 1). In an attempt to prevent further employment of this approach in method comparison studies biostatisticians repeatedly emphasized the pitfall of correlation analysis [26], [33], [59].

Although regression was proposed as a tool for the evaluation of agreement when the two methods of measurement have different units [58], it is more a calibration approach, i.e. one would try to predict the value of the standard method (N_V_) from the value obtained by the alternative method (N_A_). While regression analysis allows calculating a 95% prediction interval, something akin to the limits of agreement of the Bland-Altman analysis, it is still ‘blind’ to a systematic error, i.e. bias [33].

Thus, there is no way that would allow directly comparing the outcomes of the two designs for a single cell population.

### 2D counts of cell profiles show marked and variable deviations from 3D counts

Because the two approaches delivered data with different scale units we attempted to eliminate them by calculating a relative variable, which would be zero-dimensional and allow a direct comparison of both methods. This is represented by the ratio of CD68^+^ to CD3^+^ counts for each approach. At this point we would like to emphasize we do *not* pursue to recommend the implementation of cell density ratios in future quantitative airway biopsy research. This approach is solely meant to facilitate a sound assessment of the performance of the 2D estimator versus the 3D gold standard as recommended by the ATS and ERS [13].

As the 2D and 3D counting were performed simultaneously, i.e., on the same fields of view, one would expect the zero-dimensional ratios of macrophages to T-lymphocytes to be fairly close (accounting for the inherent random measurement error) if no bias were present. This is frequently regarded as the null hypothesis of a statistical analysis based on hypothesis testing. Besides correlation analysis this is another inappropriate approach for method comparison studies [27], [33]. A great measurement error of one or both methods would be an important confounder reducing the chance of a significant difference, without being proof of equality of the results. For demonstrative purposes only, we also adopted this null hypothesis and tested it. The ratios showed statistically significant differences between the 2D and the 3D designs (Figure 5) when counting all stained cell profiles, with the 2D approach overestimating larger cells (CD68^+^ macrophages) by the factor of 1.7 to 3.4 in the two study groups (Figure 5 and Table 3). Apart from being very pronounced, the discrepancy of the two designs is also subject to a large and significant variation (in this case twofold, P<0.005) between the different study groups. This precludes any approach to define a general ‘correction factor’ to transform the results of a 2D approach into the real 3D quantity.

### 2D counts of nuclear profiles show small but systematic and variable deviations from 3D counts

Assuming that the nucleus size varies less than the cell size, opting to count only cell transects whose nucleus appears in the section plane theoretically should reduce the size-bias [2]. When counting only macrophage profiles showing a nucleus the differences of the ratios were not large enough in either group to achieve statistical significance. However, the inability to reject the null hypothesis does *not* imply equality of results – it merely says that the difference is not large enough for significance to be achieved based on this sample size. Thus, we could not conclude that for each subject the individual ratios by each design were ‘equal’ within the tolerance for measurement error.

A simple and robust solution for the comparison of different methods was suggested by D.G. Altman and J.M. Bland more than two decades ago [33], [59]. Subsequently the Bland-Altman analysis was amended for non-uniformity and heteroscedasticity of the differences [27]. By plotting the results of the two methods against each other one can easily notice that they are widely scattered around the line of equality y = x (Figure 6). Although we can already conclude that, based on our relatively small samples, agreement of the methods is not very good, it is necessary to look at this in more detail: how large are the random differences and how acceptable is that for our purpose? Is there a systematic difference (i.e. bias) when counting nuclear profiles compared to the 3D counting using the physical disector? Moreover, if any bias is present, is it constant or proportional to the magnitude of the measurement? If no systematic error were present, the results should be alike, within the achieved precision of the measurements. In contrast to hypothesis testing, agreement is not something which is present or absent (i.e. true or false), but something which must be quantified – the decision about what is acceptable agreement is a biological one; statistics alone cannot answer such a question. For this, we need to define satisfactory agreement in advance and then verify whether most differences are smaller than our *a priori* set limits. In this case, we already set the acceptance limits for the agreement at 30% of the mean ratios, i.e.±0.12 for the non-smoker group and ±0.15 for the smoker group.

Plotting the differences of the ratios by the two methods against their means as shown in Figure 7 [33], [34] revealed a striking correlation. As already mentioned we opted for fitting a linear model to the data in the Bland-Altmann plot. For the non-smoker group the regression of the differences (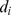) on the means (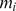) gave Eq. 1, which represents the proportional bias of the 2D ratios compared to the 3D ratios. The high statistic power of the performed regression indicates a high appropriateness in describing the relationship between the differences and the magnitude.

In the smoker group the fitted linear regression model did not reach the chosen level of significance of α = 0.05. Hence, we cannot conclude that the ratio differences in the smoker sample follow the linear distribution described by the regression equation. This can also be visualized by drawing the 95% confidence interval of the regression line – between the two curves one could also fit several horizontal lines, which would contradict a relation between the dependent variable 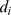 and the independent one 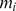. Since the statistical power of the performed regression was 43.4%, we are more likely to decide the regression does not fit the data, when the relationship described by it actually exists, than to accept it. Therefore, we can neither rely on the fitted model, nor assuredly reject it. In order to achieve a power of at least 80% with α = 0.05 and r = 0.715 we suggest to increase the sample size to n = 13 in any future investigation with a similar design. Increasing the sample size instead of improving the precision of the estimates per subject is also in accordance with the already famous dogma of stereology ‘do more, less well!’ [52]. A subsequent polynomial regression showed no fitting improvement for higher order equations, so we decided to elaborate on the linear model. The lack of statistical significance and power can also be entailed by outliers. Especially in small groups with a low variance, it is advisable to assess the impact of such outliers by eliminating them and repeating the statistical analysis [27]. By examining the plot of the ratio differences against the means, we could easily identify two large outliers (encircled in Figure 7b). As these two subjects appeared to have very low CD68^+^ and CD3^+^ N_V_ (the lowest in our sample), the counting results were very low and therefore the CE quite high in both designs. This also led to high CE_r_ (up to 25%) of the calculated ratios. As this high measurement error is likely to be a strong confounder in a sample of n = 7, we decided to exclude these two subjects and then repeat the regression analysis of the differences on the means. This led to a remarkable improvement, confirming the contribution of the independent variable (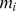) to predicting the dependent variable (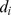).Even though acceptable agreement had to be redefined and the range became narrower, the recalculated regression based limits of agreement fully complied with this new definition.

In an eye-gauge attempt to assess the behaviour of the 2D bias in different populations, we noticed that the coefficients of the Eq. 1 and 2 appear to be similar. Subsequent formal testing revealed a significant difference between their intercepts even in our small groups. Thus, the magnitude dependent deviation of the 2D estimator from the 3D gold standard is described by a different equation in each group.

Summarizing, even though the differences between the mean ratios of N_V_ and those of N_A nucleus_ were not statistically significant and they showed a consistent correlation (Figure 5), the Bland-Altman analysis identified a non-uniform, cell density dependent bias of the 2D profile number estimator (Figures 7a and c). Hence, the agreement between 2D and 3D counting approaches is not sufficient and their results cannot be used interchangeably. The introduced bias follows different models in various groups so that a universal ‘conversion formula’ seems unattainable. We conclude that 2D counting designs are not appropriate for quantifying inflammatory cells in the airway mucosa. Counting of cell profiles clearly overestimates larger cells, thereby distorting the differential inflammatory profile to a variable and non-definable extent in different populations and/or clinical states. 2D counting of nuclear profiles failed to be reliable as well. The bias introduced by this design is not constant throughout the measurement range and therefore a general ‘correction’ cannot be applied. Consequently, we recommend using a 3D counting design in studies that aim at determining numerical densities or absolute cell numbers. Whereas in our approach comparing two different methods by using identical sections and fields of view the use of only one biopsy per subject is justified, studies aiming at the comparison of two or more different groups, the use of multiple biopsies per subject is highly recommended.
